# Detecting Heavy Metal Pollution in an Organized Industrial Zone: Soil–Plant Accumulation Patterns in a Medicinal Plant (*Calamintha nepeta* subsp. *glandulosa*) and Associated Health and Environmental Risk Implications

**DOI:** 10.3390/toxics14010089

**Published:** 2026-01-19

**Authors:** Ibrahim Ilker Ozyigit, Belma Gjergjizi Nallbani, Ibrahim Ertugrul Yalcin, Goksel Demir, Gulten Kasoglu, Bertug Sakin

**Affiliations:** 1 Department of Biology, Faculty of Science, Marmara University, 34722 Istanbul, Türkiye; 2 Environmental Issues Application and Research Center, Marmara University, 34722 Istanbul, Türkiye; 3 Faculty of Pharmacy, UBT-Higher Education Institution, 10000 Pristina, Kosovo; 4 Department of Civil Engineering, Faculty of Engineering and Natural Sciences, Bahcesehir University, 34353 Istanbul, Türkiye; ibrahimertugrul.yalcin@bau.edu.tr; 5 Department of Occupational Health and Safety, Faculty of Hamidiye Health Sciences, Health Sciences University Türkiye, 34668 Istanbul, Türkiye; 6 Department of Mathematics, Faculty of Science, Marmara University, 34722 Istanbul, Türkiye; 7 Department of Speech and Language Therapy, Faculty of Hamidiye Health Sciences, Health Sciences University Türkiye, 34668 Istanbul, Türkiye

**Keywords:** bioaccumulation, biomonitoring, Dilovasi, ICP-OES, mineral nutrient, pollution, toxic metal

## Abstract

Dilovasi district of Kocaeli is one of the largest industrial regions, and due to its high production capacity and industrial waste, the soil heavy metal levels in this region are exceptionally high. Consequently, this study focuses on essential elements (B, Ca, Cr, Cu, Fe, K, Mg, Mn, Na, Ni, Zn) and non-essential elements that are considered toxic to humans (Al, Cd, Pb), covering a total of thirteen elements. Accordingly, this study aims to highlight the degree of pollution in a Turkish Organized Industrial Zone located in the Dilovasi district of Kocaeli by quantifying the concentrations of the aforementioned elements in *Calamintha nepeta* subsp. *glandulosa* plants and soil samples, and by assessing their potential implications for human health. Significant accumulation of heavy metals in both soils and plant parts suggests that metal contamination, especially that of Fe (up to 1009.2 mg kg^−1^), is a matter of great concern in the Dilovasi district. The results revealed that the concentrations (mg kg^−1^) of Cr (23.0 ± 0.1), Fe (1292.5 ± 5.6), Pb (36.9 ± 0.1), Zn (151.2 ± 0.8), and Cd (3.6 ± 0.1) were considerably higher. However, the concentrations of Cu, Mn, and Ni were found to be within the permissible limits in accordance with the American Herbal Products Association and the World Health Organization referenced guideline values. Furthermore, heavy metal concentrations in *C. nepeta* subsp. *glandulosa* were generally higher in areas characterized by elevated soil metal levels, indicating a clear correspondence between soil contamination and plant metal content. Based on these findings, *C. nepeta* subsp. *glandulosa*, a plant with culinary and medicinal value, can be considered a useful bioindicator for assessing local heavy metal contamination.

## 1. Introduction

The evaluation of environmental pollution has historically been based on environmental monitoring, which involves examining water, air, sediments, soil, and occasionally living organisms. Within this framework, the Dilovasi Industrial Zone in Kocaeli Province is recognized as one of Türkiye’s most polluted areas, where dense metallurgical and chemical industries, combined with unfavorable topography, have led to elevated particulate matter and toxic emissions [1]. In this context, *Calamintha nepeta* subsp. *glandulosa* was selected as a possible indicator species due to its wide local distribution, traditional use by residents as herbal tea and medicine, and its physiological ability to accumulate airborne pollutants, making it both ecologically and culturally relevant for assessing environmental health [2,3]. Bioanalytical approaches, which rely on biological materials for environmental assessment (e.g., biotests and biosensors), constitute one major class of biological techniques used in environmental monitoring [4,5]. A second class is biomonitoring, which involves the planned use of biota in conventional chemical analysis and early-warning frameworks [6]. The World Health Organization emphasizes that biomonitors and bioindicators should be clearly differentiated. Specifically, biomonitors provide evidence for the presence of contaminants and can additionally inform on the magnitude and intensity of exposure, whereas bioindicators reveal the presence or absence of a contaminant through characteristic symptoms or measurable biological responses [7,8,9,10]. Careful methodological considerations are essential for interpreting biomonitoring data accurately. Nevertheless, biomonitoring strengthens our understanding of the potential impacts of chemicals on human health and the environment and supports discussions on risk-reduction strategies, including (1) awareness raising, (2) no immediate action, (3) controlling substance exposure, (4) banning substances, (5) proxy monitoring, (6) intensified monitoring efforts, and (7) further research [3,11,12].

Because metals can mimic essential ions or inhibit enzymes, they can be hazardous to living things. This is contingent upon their oxidation state, capacity to form complexes, and concentration. Since inorganic metal compounds are readily soluble and may enter organisms across cell membranes with ease, they have the most toxic qualities [13]. Metals are generally classified as essential, with vital biological functions, or non-essential, which lack physiological roles and may be toxic [14]. While non-essential elements have no known biological role and cause toxicity by competing with essential metals for positions on active enzymes or membrane proteins, essential metals and micronutrients are necessary for the efficient functioning of biological and metabolic processes in animals. However, when exposed to high concentrations, physiologically necessary metals have the potential to be hazardous to people and other living things. Living things may absorb and accumulate heavy metals in addition to vital nutrients [15,16]. Another crucial component of monitoring research is biomonitoring of heavy metals, and a plethora of papers have demonstrated the significance of plants and animals in these investigations [17,18].

The environment is currently exposed to a variety of pollution sources, including anthropogenic activities and industrial pollution caused by humans. High quantities of heavy metals, including Cd, Hg, and Pb, are found in soils, rocks, and sediments and occur naturally in the earth’s deep strata [19,20]. Additionally, significant air, water, and soil pollution can result from anthropogenic effects of heavy metals, which are mostly connected to extraction, mining, and refining processes. After being discharged into the air, water, or soil, heavy metals do not degrade; instead, they build up in soils, sediments, and biota [16,21,22].

Thus, heavy metal deposition in the atmosphere has surpassed upper limit values due to excessive levels of air pollution in industrial regions. Given its significant industrial density and the proximity of two of Türkiye’s busiest roads (O4 and D-100) to its center, the Dilovasi Organized Industrial Zone (DOIZ) in Kocaeli, Marmara Region, is among the most polluted districts. In the last ten years, 15% of Türkiye’s industrial sector has been based in Kocaeli City [23]. In the DOIZ, there are 229 companies working in 35 distinct industries, the bulk of these companies are quarries and chemical and metal (aluminum, iron–steel, and smelting) factories [24].

The present study investigates the extent of environmental pollution in the Dilovasi district, an area characterized by intense urban and industrial activity, by integrating plant- and soil-based evidence. In this context, *C. nepeta* subsp. *glandulosa* and co-located soil samples were analyzed to assess the distribution of accumulated heavy metals and to evaluate potential human health risks associated with environmental exposure. Ultimately, the primary objective of this research is to assess whether the locally collected plant material can be safely consumed for food or medicinal purposes given the contamination profile of the region.

## 2. Materials and Methods

### 2.1. Description of Location, Samples, and Species

Istanbul, located at 41°00′49″ N and 28°57′18″ E, lies across the Bosphorus Strait between Europe and Asia. The city has a transitional Mediterranean–oceanic climate, with cool, wet winters, warm to hot summers, and annual precipitation of about 680 mm, influenced by its maritime position and varied topography. Consequently, the state of the roadway transportation has become more significant. D-100 and TEM highways pass through Kocaeli; Yalova and Bursa cities are connected to Kocaeli by the D-130 highway. Despite being one of the smallest districts of Kocaeli, Dilovasi has one of the greatest concentrations of businesses. The main industrial activities in DOIZ comprise the manufacture of chemicals, food and beverage products, plastics, textiles, wood and forest products, petrochemicals, metals, hardware, glass, electrical and electronic equipment, wire rope, machinery, and metal heat-treatment processes [24,25]. Due to these elements, the DOIZ has a significant chance of producing air pollution from industrial, automotive, and urban sources. The DOIZ’s rocky topography and bowl-shaped terrain also prevent the area’s air from mixing adequately, which causes inversion and very polluted air [3,26].

Hence, Basibuyuk Forest, situated in the Maltepe district on the Anatolian side of Istanbul in northwestern Türkiye, represents an important green area within the Marmara Region. The forest is characterized by diverse vegetation and plays a significant role in maintaining local ecological balance, providing habitat for various plant and animal species while also serving as a recreational area for surrounding communities. Situated approximately 25–30 km from the Dilovasi district, the forest is geographically close to one of the most industrialized areas of Kocaeli Province, making it a valuable reference site for comparative environmental monitoring. In addition to its ecological importance, Basibuyuk Forest contributes to air quality improvement and soil protection, and its underlying Paleozoic metamorphic basement rocks overlain by Mesozoic sedimentary sequences reflect the complex geological history of the Pontide tectonic belt [27,28].

In this study, plant materials were collected as true biological replicates. Specifically, eight independent individual plants were sampled at each of five distinct locations, and corresponding soil samples were collected from the immediate vicinity of each individual. Location 1 is characterized by a highway transportation station. Location 2 is defined as Ballikayalar Valley. Ballikayalar Natural Park is near the village of Tavsanli in Kocaeli. There are several little waterfalls, lakes, and deep fissures in the hillside, which is a steep and narrow valley spanning between 40 m and 1.5 km. It is characterized by diverse types of animals and birds.

Location 3 is defined as the Brook Coast. Location 4 is characterized by a railway station, through which millions of people have been transported by train to date. Furthermore, investigations by district and metropolitan municipalities are being conducted with the goal of expansion. Additionally, location 5 is distinguished by its dense industrial area, with tire and rubber production facilities, the automotive industry, and iron and steel manufacturing. Basibuyuk Forest was defined as the control location, as it is a region independent of industrial activity and human population.

*C. nepeta* subsp. *glandulosa* is a semi-evergreen perennial herb that belongs to the mint family and is also known as lesser calamint. The plant is native to Europe and the Mediterranean region. The plant has a compact, mounded structure and grows up to 30 cm (12 inches) tall. It has small, hairy, ovate, toothed, mintily fragrant, dark green leaves beneath a cloud of long-lasting, branched, airy, whorled spikes of small, soft blue/lilac to white, two-lipped flowers borne from July to September. It is an excellent edging plant for walks, patios, or herb gardens and is also effective when sprawled over low retaining walls or grown in containers [29].

Due to its antibacterial, antioxidant, and anti-inflammatory properties, as well as its stimulant, digestive, tonic, and antiseptic properties, *C. nepeta* subsp. *glandulosa* is utilized in Mediterranean traditional medicine [30]. Additionally, this plant exhibits heat tolerance and drought resilience, two highly significant traits for its survival in Mediterranean regions, where it is utilized to reduce soil erosion and enhance soil quality [29].

### 2.2. Sample Preparation

Plant parts and soil samples were collected from five locations within the DOIZ and its surrounding areas, including a control site, and transported to the laboratory in sterile bags. Sampling was conducted during the spring vegetation period (May), yielding a total of 48 independent individual plants (n = 8 individuals per location) and 48 paired soil samples (n = 8 per location), with each soil sample collected from the immediate vicinity of the corresponding sampled plant (paired design; no compositing). At each designated soil sampling point, approximately 500 g of soil was collected from the 0–20 cm topsoil layer, placed in sterile bags, and transferred to the laboratory. Soil samples were transferred into glass Petri dishes and dried at 80 °C for 48 h; they were then passed through a steel sieve with a 2 mm mesh size to prepare them for weighing and subsequent digestion.

From each individual plant, stem and rhizome tissues were collected as single, separate matrices, resulting in 48 stem samples (n = 8 per location) and 48 rhizome samples (n = 8 per location). To evaluate the potential influence of atmospheric deposition on foliar elemental profiles, leaves were collected in sufficient quantity from each individual (typically 8–10 leaves per plant) and divided into two equal sub-samples from the same plant (paired within-plant design). One leaf sub-sample was rinsed three consecutive times with ultrapure deionized water (≥13 MΩ·cm), each rinse lasting approximately 30 s with gentle manual agitation; no detergents or surfactants were used. The second leaf sub-sample was processed without any rinsing step to retain potential surface-associated material. After the rinsing step (where applicable), leaf material was allowed to drain briefly, and all plant materials (washed leaves, unwashed leaves, stems, and rhizomes) were oven-dried at 80 °C for 48 h. Dried samples were cooled in a desiccator prior to weighing, milled to a fine powder using a clean, acid-washed micro-hammer mill, and passed through a 1.5 mm sieve to ensure homogenization before digestion.

For elemental analysis, approximately 0.200–0.500 g of each dried, homogenized plant powder (dry weight) and an appropriate mass of the prepared soil fraction were weighed into acid-cleaned digestion vessels. Samples were digested with 5–8 mL of ultrapure HNO_3_ following a brief pre-digestion period at room temperature and then subjected to microwave or hotplate digestion at 160–180 °C for 20–40 min until clear solutions were obtained; when necessary, 1–2 mL of H_2_O_2_ was added to complete organic matrix breakdown. After cooling, digests were quantitatively transferred to volumetric flasks and diluted to 25–50 mL with 1–2% HNO_3_ to ensure matrix compatibility with ICP-OES calibration standards. Where particulates remained, solutions were clarified by filtration through 0.45 µm membranes or by centrifugation at 3000–5000 g for 10–15 min, and the supernatant was collected for analysis. Inductively coupled plasma optical emission spectroscopy (ICP-OES) was used to quantify Al, B, Ca, Cd, Cr, Cu, Fe, K, Mg, Mn, Na, Ni, Pb, and Zn in the digested soil and plant matrices [3,31].

### 2.3. Accuracy of Data and Determination of Element Contents

All concentrations were measured with an exceptionally low margin of error of 0.72–1.19% (RSD) using previously developed calibration standards (Table 1). A Microwave Digestion System (Berghof MWS2) and the EPA 3051A analytical method for ICP-OES were used to dissolve plant samples. Plant samples were digested by adding 8 mL of concentrated nitric acid (65%, Merck) into Teflon digestion vessels. In contrast, soil samples were treated with a mixed acid solution consisting of 6 mL of nitric acid (65%, Merck), 3 mL of hydrochloric acid (37%, Merck), and 2 mL of hydrofluoric acid (48%, Merck) to ensure complete matrix decomposition. The elemental composition of the samples was examined using a PerkinElmer-Optima 7000DV ICP-OES [32]. Calibration curves were generated using calibration standards that were prepared at eight different concentrations (0–25–50–100–250–500–750–1000 ppb) for each element; yielding R^2^ > 0.999 (Table 1). Additionally, during the analytical process, selected calibration standards were intermittently measured to verify instrumental stability and ensure data consistency throughout the run. The Limit of Detection (LoD) and Limit of Quantification (LoQ) values for each of element were discovered by looking at blank solutions (Table 1) [33,34]. To determine the samples’ elemental compositions, spectral lines were chosen according to the pertinent literature, as presented in Table 1 [34,35,36].

The Recommended Daily Allowance (RDA) (%) (100 g/dw) values for Ca, Cu, Fe, K, Mg, Mn, Na, and Zn elements were calculated to reveal the Dietary Reference Intake potentials of *C. nepeta* subsp. *glandulosa* when utilized as a culinary herb, spice, or tea, as evaluated in this study [16,17]. The target hazard quotient (THQ), estimated daily intake (EDI), and hazard index were applied to ascertain exposure to Cd, Cr, Ni, and Pb, which may endanger the lives of consumers who ingest plant leaves (HI) [37,38,39]. The quantitative evaluation of the potential non-carcinogenic effects of each hazardous metal was performed using THQ [36]. Hence, the potential risks to general health from exposure to certain hazardous metals were predicted using the hazard index (HI).

Following the determination of elemental concentrations in soil and different plant parts, bioaccumulation and translocation indices were calculated to assess the uptake efficiency and translocation of elements within *C. nepeta*. The bioaccumulation factor (BAF) was calculated as the ratio of elemental concentration in the rhizome to that in the corresponding soil, providing an estimate of the plant’s capacity to accumulate elements from the surrounding environment. In addition, the translocation factor (TF) was determined as the ratio of elemental concentration in the washed leaf to that in the rhizome, enabling evaluation of the efficiency of element transfer from underground parts to above-ground tissues. These indices were applied following established methodologies [40,41,42] and are presented in Table 2 to elucidate species-specific accumulation and translocation behaviors.

### 2.4. Statistical Evaluation of Metal Concentrations

All element concentration calculations were performed considering the dry weight of soil, rhizome, stems, and leaves. Multivariate analysis of variance (MANOVA) was performed, and where appropriate, group means were compared using Tukey’s HSD post hoc test in IBM SPSS Statistics v25 (IBM Corp., Armonk, NY, USA). Levels of statistical significance were denoted as * *p* < 0.01 and ** *p* < 0.05, with a two-tailed test employed for all analyses.

## 3. Results and Discussion

### 3.1. Elemental Concentrations in Plant Parts and Soils Across Locations

Mean concentrations of elements in the leaves, stem, rhizome, and soil of *C. nepeta* subsp. *glandulosa* across five locations, including the control location, are shown in Table 3. MANOVA was performed; when appropriate, Tukey’s HSD was used for multiple comparisons., and the observed differences were found to be statistically significant and internally consistent at the defined significance levels (*p* < 0.05 and *p* < 0.01). Soil qualities are vital in food production, and heavy metal contamination of this critical resource, as well as their subsequent absorption and bioaccumulation in food crops, poses significant environmental and health implications, particularly in developing nations. Heavy metal concentrations are controlled by soil type, pH, plant genotype, and their interactions [43,44].

Metal concentrations in soil ranged from (in mg kg^−1^) 24.4–347.6 (Zn), 26.1–190.2 (Pb), 0.5–5.6 (Cd), 3.5–48.1 (Ni), 5383.4–8087.6 (Fe), 73.9–268.1 (Mn), 11.0–64.5 (Cr), 2746.9–4227.3 (Mg), 23075.9–40611.3 (Ca), 11.6–65.8 (Cu), 8081.9–15898.4 (Al), 1500.1–3634.7 (Na), 14567.0–26475.8 (K), and 9.8–99.0 (B). The lowest values were detected at the control location, as the control location is devoid of industrial facilities, except for K, for which the lowest value was detected at location 3.

Furthermore, Cd, Cr, Ni, and Mg reached their highest concentration in the soil of location 1, while Pb reached its highest concentration in the soil of location 2. In addition, the soil at location 3 had the highest concentrations of Cu, Na, and B, whilst the soil at location 4 had the highest concentrations of Fe, Mn, Ca, and Al. Finally, Zn and K reached their highest concentrations in the soil of location 1.

The distribution of the highest values across multiple locations suggests that the district is influenced by considerable and spatially heterogeneous pollution inputs. Heavy metals are responsible for many health consequences; for example, carcinogenicity is encouraged by excessive exposure to certain Cr species (e.g., Cr(VI)) [45,46], and children poisoned by Pb experience irregularities in intellectual development [47]. In addition, several human organs such as the kidneys, brain, liver, skin, and hearth may be adversely affected by heavy metal exposure. Therefore, risk-reduction measures (e.g., emission control, exposure minimization, and routine monitoring) are warranted in areas with elevated heavy metal pollution [48,49].

Heavy metals enter plants through both leaves and roots. Nonetheless, it is difficult to tell whether heavy metals in plant organs come from soil or the environment, because intake pathways from soil and air may occur simultaneously. Furthermore, leaves are the most heavily contaminated plant organs, as they may absorb heavy metals from the air through stomata during photosynthesis [50,51]. The xylem carries water and dissolved minerals from the roots to the stem and leaves. The water-conducting elements of this tissue, which continue uninterrupted from the roots to the leaves, are the trachea and tracheids [52]. Hence, leaves are considered the most appropriate body part for tracing the contamination of heavy metals in the air [43].

For all elements analyzed within the scope of this study, the highest values were recorded in unwashed leaves. For Zn, Pb, Cd, Ni, Fe, Mn, Cr, Mg, Ca, Cu, Al, Na, K, and B, the values (in mg kg^−1^) were found as 151.2 ± 0.8 (location 4), 36.9 ± 0.1 (location 3), 3.6 ± 0.1 (location 5), 24.6 ± 0.2 (location 5), 1292.5 ± 5.6 (location 1), 171.7 ± 4.6 (location 4), 23.0 ± 0.1 (location 1), 2582.3 ± 63.9 (location 2), 9979.6 ± 118.0 (location 4), 29.2 ± 0.2 (location 5), 733.6 ± 7.5 (location 4), 966.7 ± 4.4 (location 3), 6780.1 ± 87.1 (control location), and 31.9 ± 0.1 (location 3), respectively. The fact that high values are distributed across different locations, rather than in a single location, indicates that pollution in the region is on a significant scale, considering that the expressway and railway stations are in locations 1 and 4, respectively. However, location 5 is distinguished by pronounced industrial activities, including tire and rubber production facilities, the automotive industry, and iron and steel manufacturing. Surprisingly, the highest values of Mg and B are evaluated at locations 2 and 3, which are defined as Ballikayalar Valley and Brook Coast, respectively.

Cr enters our environment through the combustion of coal and oil and it is discharged into the environment through drainage and fertilizers. It is also present in petroleum, colors, oxidants, catalysts, and compost [53]. Humans can be exposed to chromium by inhalation, skin contact, drinking, and ingesting chromium and its compounds. In this study, the lowest Cr value was found to be 2.4 ± 0.1 mg kg^−1^ (w leaf) in location 5. Mn is involved in several physiological processes in humans and other living beings and is the most important component of enzymes [54]. Although it is less hazardous than other heavy metals, an increase of more than 0.1 mg L^−1^ in its content in household water causes taste changes and sediment/precipitation problems. Increased manganese concentrations over 0.24–0.35 mg L^−1^ may induce transient concentration failure in youngsters. A study found that children who drink water with a high Mn content had poor attention and attentiveness in school [55]. The World Health Organization (WHO) guideline for manganese in drinking water previously derived as 0.4 mg L^−1^ (400 µg L^−1^), while the U.S. Environmental Protection Agency (EPA) has set a secondary maximum contaminant level (SMCL) of 0.05 mg L^−1^ (50 µg L^−1^), primarily for esthetic concerns, such as taste, color, and staining. As is well known, when *C. nepeta* subsp. *glandulosa* is prepared as an herbal tea, a fraction of leaf-associated Mn may leach into the infusion and contribute to the Mn level of the beverage as well as other co-occurring metals; therefore, drinking-water values are cited here only to contextualize the tea-consumption pathway. Within the scope of this study, the lowest Mn value was found to be 5.3 ± 0.1 mg kg^−1^ (stem) in location 2. Fe is a necessary component for all living organisms, including humans. Fe is also a basic component of proteins and several enzymes, including hemoglobin and myoglobin, as well as a necessary component for the growth and survival of nearly all living species [56,57,58]. Consuming excessive levels of Fe have been linked to hemochromatosis, a hereditary condition. The US EPA reports that drinking water with a high Fe concentration does not necessarily have a detrimental health impact, because individuals acquire increased Fe concentrations mostly via the consumption of Fe-rich foods [59]. In the framework of this study, the lowest Fe value was found to be 203.8 ± 8.7 mg kg^−1^ (stem) in location 2. Ni is a common element that may be found in nature as metal oxides or metal sulfates, with NiSO_4_ (nickel sulfate) found in soil. Ni atoms settle in the soil from the air or are washed out of the air by rain. Nickel and its compounds, which come from the refining processes and refining nickel, are carcinogenic elements [60]. According to published research, the carcinogenic potential of Ni compounds varies by chemical form and exposure route; therefore, generalizations based solely on water solubility should be avoided [55]. Within the parameters of this investigation, the lowest Ni value was found to be 6.2 ± 0.1 mg kg^−1^ (rhizome) in location 2. Cu is necessary for the development of connective tissue, nerve coverings, and bones, and it collaborates with Fe in energy metabolism. Because of its availability, Cu is used in a variety of industries and agriculture. The human body obtains Cu from eating, drinking, and breathing. Cu at high levels is detrimental to human health. The presence of Cu at the air limit and its adverse effect on human health results from inhaling, particularly for individuals who live near metal smelting sites. Metal fume fever is caused by inhaling copper vapors [55,61]. Within the scope of this study, the lowest Cu value was found to be 16.7 ± 0.1 mg kg^−1^ (stem) in location 2.

Agricultural and industrial sources can release Cd, a hazardous metal that is found in the environment naturally. The majority of non-smokers obtain their Cd consumption mostly via food. The kidneys are an effective storage sites of Cd, with a half-life of 10–30 years, and its concentration is proportional to urine (U-Cd) [62]. Furthermore, individuals exposed to higher doses of Cd may have elevated death rates and cancer risks, according to current findings [48,63]. In this study, the lowest Cd value was found to be 0.8 ± 0.1 mg kg^−1^ (w leaf) in location 2.

Anthropogenic emissions are responsible for around 13% of the Al in the atmosphere. The main human sources of particulate matter containing Al include the burning of coal, the manufacture of aluminum, iron, and steel foundries, refineries that produce brass and bronze, as well as motor vehicle emissions. For instance, smoke from cigarettes may have an impact on the amount of Al in the air. Al builds up in endocrine glands and damages them via oxidative stress; as a result, the number of hormones released into the circulation to work on target organs is reduced, leading to organ hypofunction [64]. Within the parameters of this investigation, the lowest Al value was found to be 43.6 ± 0.1 mg kg^−1^ (stem) in location 2.

Individuals who are exposed to Pb as a contaminant include automobile drivers, particularly in congested areas. All pollution states result from inhalation, ingestion of products contaminated with lead, or the use of cosmetics containing lead in their composition. In the framework of this study, the lowest Pb value was found to be 5.0 ± 0.1 mg kg^−1^ (stem) in location 4. The finding of low values mainly in locations 2 and 4 suggests that these areas are comparatively less polluted, considering the previously described data. Thus, it can be concluded that the overall metal profile of leaves closely paralleled that of the soil fraction. The average values of Cd, Cr, Cu, Fe, Mn, Pb, Zn, and Ni in plants, as well as the values accepted as toxic, are presented in Table 4. In addition, acceptable/normal limits for mineral nutrients in plants, as described int the relevant literature [65,66,67,68], are as follows: Al ranges from 15 to 100 (mg kg^−1^), B ranges from 3.0 to 90 (mg kg^−1^), Ca ranges from 300 to 30,000 (mg kg^−1^), and K ranges from 1000 to 10,000 (mg kg^−1^), respectively. Within the range of reference values specified in the literature (Table 4), even the highest concentrations of Zn, Mn, and Cu are within acceptable limits, while Cd, Ni, and Cr exceed acceptable values but have not reached toxic levels. Additionally, Pb and Fe values are included within toxic ranges. Considering mineral nutrients, Al has exceeded the expected limits, whilst B, Ca, and K are within normal ranges [65,69,70].

### 3.2. Nutritional Significance, Heavy Metal Accumulation Patterns, and Associated Health Risks of Calamintha nepeta subsp. glandulosa

In the current study, the values were also compared with the recommended values of the American Herbal Products Association (AHPA) in collaboration with the World Health Organization. The results revealed that the concentrations of Cr, Fe, Pb, Zn, and Cd were shown to be considerably higher than the permissible limits determined by FAO/WHO and AHPA [71]. However, the concentrations of Cu, Mn, and Ni were found to be within the permissible limits in accordance with the American Herbal Products Association (AHPA) and WHO (2025) referenced guideline values (Table 4).

In a research study conducted in Türkiye, the concentrations of Cu, Fe, and Zn in the leaves of some roadside native plants, including *C. nepeta* subsp. *glandulosa,* were determined around “polluted (near highways)” and “non-polluted (far from highways)” sites near Ordu province during summer of the year 2006. Regarding heavy metals, there were notable distinctions between “polluted” and “non-polluted” places. Considering the data from polluted areas, Cu and Fe levels in *C. nepeta* subsp. *glandulosa* were found to be considerably lower, while Zn value was found to be higher than those reported in the current study [72]. Furthermore, in a study performed around the Barbadalhos Pb mine in central Portugal, although significant accumulation of heavy metals in the soils and flora of *C. nepeta* subsp. *glandulosa* was reported, Cr, Cu, Fe, and Ni levels were found to be lower than those reported in our study, whilst Pb and Zn values were found to be higher. The higher detection of Pb values is compatible with the evaluation of the study in the Pb mine [73]. Moreover, a study conducted in the Croatian National Park in Northern Velebit aimed to determine the elemental composition of different indigenous medicinal plants, including *C. grandiflora,* together with soil samples. All examined elemental values in *C. grandiflora* were discovered to be lower than those observed in our investigation, except for Mg and Mn, where the detected values were found to be higher than this study [74].

Additionally, in research performed at a mine in Rosalgar (Setúbal district, Portugal), three different Lamiaceae species, including *C. nepeta* subsp. *Glandulosa,* were selected to determine their elemental compositions. According to the values recorded for *C. nepeta* subsp. *glandulosa*, K, Mg, and Mn values were found to be higher than those values detected in the current study, while the other values were found to be lower than in the present study [29]. Compared to the aforementioned literature values, the results suggest that contamination pressure in the region is relatively high (Table 5).

To evaluate soil-to-plant transfer and internal redistribution of elements within plant tissues, BAF and TF were calculated (Table 2). The BAF, defined as the rhizome-to-soil concentration ratio, varied notably among elements and between the control and industrial zones. At the control site, BAF values exceeded 1 only for Ni and B, indicating selective accumulation, whereas all other elements exhibited values below unity, reflecting limited rhizome uptake. In contrast, in the industrial zone, BAF values remained below 1 for all elements, suggesting an apparent limitation of root-mediated metal uptake under elevated metal loads.

The TF values, calculated as the ratio of washed leaf concentration to rhizome concentration, displayed element-specific and site-dependent patterns. At the control site, TF values exceeded 1 for Zn, Fe, Mg, Ca, Na, K, and B, indicating effective translocation to aerial tissues. In *C. nepeta*, where leaves are directly consumed, assessing TF based on a rhizome-to-washed leaf comparison provides a more realistic perspective of potential dietary exposure. In the industrial zone, translocation efficiencies increased for most elements, particularly Zn, Pb, Ni, Fe, Mn, Cr, Na, K, and B, indicating enhanced internal mobility of metals under industrial stress conditions.

These findings highlight the importance of evaluating element dynamics not only in terms of root uptake but also with respect to their redistribution to edible plant parts, especially in medicinal species widely used in phytotherapy. Overall, the calculated BAF and TF values indicate that *C. nepeta* subsp. *glandulosa* does not function as a strong accumulator, as rhizome enrichment relative to soil concentrations remained below unity for most elements, particularly in the industrial zone. However, the pronounced translocation toward above-ground tissues (TF > 1 for multiple potentially toxic elements and essential nutrients), together with the strong correspondence between leaf concentrations and site-specific pollution intensity, supports its role as an integrative bioindicator of local metal contamination. In this context, *C. nepeta* primarily reflects the combined effects of soil-derived uptake and atmospheric deposition rather than exhibiting a quantitative accumulation capacity.

In order to estimate potential health risks associated with exposure to substances in the evaluated plant samples, the estimated daily intake of metals (EDI) was investigated. The EDIs of heavy metals for Turkish people through consumption of *C. nepeta* subsp. *glandulosa* from selected locations are shown in Table 6. To calculate the non-carcinogenic risk level resulting from pollution exposure, especially heavy metal exposure, the target hazard quotient (THQ) was utilized. The results of our study showed that THQ and HI values were lower than 1, indicating that the population of the DOIZ consuming edible *C. nepeta* subsp. *glandulosa* plants are assumed to be at minimal risk of non-carcinogenic harmful effects. In the present study, THQ and HI values were lower 1, suggesting a low non-carcinogenic risk under the assumed consumption scenario. Nevertheless, because some elemental concentrations were relatively elevated compared with guideline-based benchmarks and literature reference values, caution is warranted, particularly under frequent or long-term use or when consumed in concentrated preparations; however, typical intake amounts for medicinal plants are generally much lower than the 100 g dry-weight basis used for RDA calculations, which substantially reduces actual exposure. While some medicinal plant species exhibit notably high Recommended Dietary Allowance (RDA) percentages for various elements, these values are calculated based on 100 g of dry plant material, a quantity far exceeding typical daily use. In reality, such plants are generally consumed in much smaller amounts, often as teas, spices, or supplements, thereby substantially reducing actual intake. Therefore, high RDA values should not be interpreted as immediate indicators of excessive intake under conventional consumption practices. Nevertheless, for plant species with exceptionally elevated elemental concentrations, these findings warrant careful consideration. They underscore the importance of informed use, especially when such plants are ingested regularly, in concentrated extracts, or in formulated dietary supplements, where elevated elemental loads may pose cumulative health risks and should be approached with due care and, where necessary, in accordance with regulatory guidance.

Accordingly, it is important to point out that THQ/HI values below 1 (low non-carcinogenic risk) does not fully exclude concerns under frequent/long-term use, sensitive groups, or concentrated preparations. Thus, intake of *C. nepeta* subsp. *glandulosa* plants selected from five different locations warrant cautious interpretation for potential consumers in the DOIZ area. However, from the data presented, consumers should pay attention to excessive heavy metal accumulation in *C. nepeta* subsp. *glandulosa* plants due to concentrations exceeding WHO/FAO limits. The RDA is the average daily level of nutrient intake that is sufficient to meet the nutrient requirements of nearly all healthy individuals in a particular life stage and gender group. RDA values vary by age, gender, and other factors, and are established by the Food and Nutrition Board of the National Academies of Sciences, Engineering, and Medicine. They are often used to plan nutritionally adequate diets for individuals [75]. Consequently, the RDA % values (100 g dw^−1^) for Ca, Cu, Fe, K, Mg, Mn, Na, and Zn were determined in *C. nepeta* subsp. *glandulosa* to support a comparative analysis of nutritional values gathered from the DOIZ and its environs (Table 7). Exceedingly high RDA percentages for elements such as Cu, Fe, and Mn were found in *C. nepeta* subsp. *glandulosa* leaves, particularly under the hypothetical assumption of a 100 g dry weight daily intake. In particular, for samples collected from the Dilovasi region, the calculated RDA values for these three elements markedly exceed 100% of the daily recommended intake. In practice, this plant is traditionally consumed in significantly smaller quantities, typically as an herbal tea, condiment, or flavoring agent. Such use rarely exceeds 2–5 g per day, rendering the 100 g intake scenario largely symbolic. Moreover, daily nutritional needs for essential minerals are generally met through a diverse diet, and the role of medicinal plants like *C. nepeta* is generally supplementary. Their value lies not in primary mineral content but in providing trace elements alongside bioactive phytochemicals that enhance overall dietary quality. Thus, while RDA-based calculations are analytically useful, they should not be interpreted as a direct nutritional recommendation, but rather as an indication of the plant’s micronutrient richness under maximal exposure scenarios.

For all elements, higher concentrations were detected in unwashed leaves. Consistently across all elements, the smallest differences were observed at the control location, supporting the interpretation that this site is comparatively less affected by airborne deposition. A more detailed assessment indicates that, for the locations showing the highest contrasts, the percentage differences between washed and unwashed leaves can be ranked as follows: Zn (location 4) 31.74%, Pb (location 3) 38.21%, Cd (location 5) 63.89%, Ni (location 5) 12.6%, Fe (location 1) 21.92%, Mn (location 4) 39.94%, Cr (location 1) 33.04%, Mg (location 2) 19.39%, Ca (location 4) 11.05%, Na (location 3) 25.53%, K (location 1) 19.87%, and B (location 3) 20.69%. These findings indicate that leaf washing substantially influences the measured elemental concentrations, underscoring the importance of this methodological step in accurately assessing atmospheric deposition and pollution levels.

In a separate nutritional perspective, a daily intake of 100 g of *C. nepeta* subsp. *glandulosa* meets more than 1000% of the Cu, Fe, and Mn requirements and more than 100% of the Zn requirement (a hypothetical scenario used for standardized comparison). However, it has been predicted that over 60% of the world’s population is deficient in Zn and over 30% in Fe. Furthermore, high Ca, Mg, and Cu deficiencies are common in both developed and developing countries [39]. These findings demonstrate that, as long as the concentrations of heavy/toxic metals are within relevant guideline-based benchmarks reported for herbal materials, *C. nepeta* subsp. *glandulosa* may be assessed as a dietary supplement due to its mineral and nutritional content. Thus, the RDA results suggest a potentially high contribution for essential elements, including Cu, Fe, Mn, Ca, and Mg in *C. nepeta* subsp. *glandulosa* plants gathered from the current locations. The distribution of the highest RDA values across different locations again emphasizes that regional pollution is significant. Therefore, use of this plant as food or medication should be approached with caution, particularly for frequent use or concentrated preparations.

Finally, analyzing the concentrations of elements found in *C. nepeta* subsp. *glandulosa*, it is concluded that for Mn, Cr, Ca, Al, Na, and B elements, respectively, the highest values detected in the plant parts were also determined in the soil samples in parallel. This suggests that classifying *C. nepeta* subsp. *glandulosa* as a moderate biomonitor species would be reasonable.

## 4. Conclusions

The findings of this study indicate that the Dilovasi district is characterized by considerable metal pollution, as evidenced by elevated concentrations detected in both soils and different plant organs. Among the analyzed elements, Fe exhibited particularly high levels, which may be attributed to intensive industrial activities and atmospheric deposition commonly associated with organized industrial zones. The observed variability in metal concentrations across sampling sites further suggests that the relative contributions of different contamination sources vary spatially within the district.

Medicinal plants themselves are not primary source of environmental contamination; however, they are highly susceptible to metal accumulation when grown or collected from polluted environments. Accordingly, herbs harvested from contaminated areas, especially when improperly handled or stored, may pose health risks rather than providing expected therapeutic benefits. This finding underscores the importance of considering the environmental context in which medicinal plants are collected.

The distribution of metals among the leaves, stems, and rhizomes of *C. nepeta* subsp. *glandulosa* revealed clear organ-specific accumulation patterns closely linked to local environmental metal loads. Such patterns suggest that plant tissues effectively reflect spatial variations in pollution intensity across different locations in the Dilovasi district, consistent with observations reported for other medicinal and aromatic plant species in contaminated environments.

When compared with values reported in the literature for *C. nepeta* subsp. *glandulosa*, the metal concentrations detected in this study, including those measured in soils, were markedly higher, indicating that the Dilovasi region is subject to substantial environmental pollution. Based on these findings, caution is strongly recommended when collecting *C. nepeta* subsp. *glandulosa* for consumption and harvesting from areas distant from industrial activities should be prioritized. Although the estimated non-carcinogenic risk levels were generally low, the elevated accumulation of heavy metals associated with the organized industrial zone suggests that potential long-term health implications cannot be ruled out, particularly under conditions of chronic exposure.

## Figures and Tables

**Table 1:** Parameters of the analytical method for ICP-OES.

Element	Spectral Line (nm)	LoD (mg kg^−1^)	LoQ (mg kg^−1^)	RSD (%)	R^2^
Al	396.153	0.531	1.770	0.84	0.999905
B	249.677	0.342	1.140	0.73	0.999951
Ca	317.933	0.984	3.280	0.95	0.999872
Cd	228.802	0.008	0.027	1.05	0.999931
Cr	267.716	0.009	0.030	1.19	0.999895
Cu	327.393	0.034	0.113	0.91	0.999927
Fe	238.204	0.462	1.540	0.72	0.999919
K	766.490	0.715	2.383	0.86	0.999884
Mg	285.213	0.401	1.337	0.93	0.999953
Mn	257.610	0.076	0.253	0.77	0.999882
Na	589.592	0.508	1.693	0.91	0.999896
Ni	231.604	0.017	0.057	1.07	0.999873
Pb	220.353	0.011	0.037	0.88	0.999867
Zn	213.857	0.079	0.263	0.96	0.999932

LoD: limit of detection; LoQ: limit of quantification; RSD: relative standard deviation; R^2^: determination coefficient.

**Table 2:** Bioaccumulation and translocation factors (BAF and TF) in *C. nepeta*.

Elements	BAF–Control	BAF–Organized Industrial Zone	TF–Control	TF–Organized Industrial Zone
	**R/S**	**L/R**	**R/S**	**L/R**
Al	0.05	0.05	0.39	0.56
B	1.18	0.19	0.19	1.25
Ca	0.09	0.20	1.91	0.96
Cd	0.94	0.55	0.90	0.46
Cr	0.37	0.13	0.81	2.24
Cu	0.46	0.38	0.64	0.83
Fe	0.02	0.05	1.60	2.64
K	0.23	0.18	1.03	1.41
Mg	0.21	0.51	1.53	0.71
Mn	0.39	0.20	0.30	2.00
Na	0.10	0.07	1.02	2.68
Ni	1.62	0.24	0.89	2.16
Pb	0.27	0.09	0.34	1.46
Zn	0.53	0.20	1.19	1.68

S: soil; R: rhizome; L: leaf (washed leaf).

**Table 3:** Concentrations of Al, B, Ca, Cd, Cr, Cu, Fe, K, Mg, Mn, Na, Ni, Pb, and Zn (mg kg^−1^) in parts of *Calamintha nepeta* subsp. *glandulosa* and co-located soil samples.

*Calamintha*		Washed Leaf	Unwashed Leaf	Stem	Rhizome	Soil
Al	Control	148.077 ± 0.861 **	230.208 ± 5.114 **	46.614 ± 0.714 **	376.352 ± 7.874 **	8081.867 ± 26.044 *
	1.loc	461.353 ± 5.443 **	540.086 ± 6.743 **	61.389 ± 0.271 *	679.034 ± 6.207 **	13,378.080 ± 44.413 *
	2.loc	301.264 ± 4.046 **	460.495 ± 5.456 **	43.580 ± 0.146 *	588.445 ± 5.037 **	11,914.507 ± 73.735 *
	3.loc	338.431 ± 7.913 **	573.058 ± 7.550 **	87.248 ± 0.115 *	659.486 ± 6.498 **	15,354.613 ± 74.541 *
	4.loc	439.208 ± 6.060 **	733.634 ± 7.520 **	77.707 ± 0.710 **	841.660 ± 10.229 **	15,898.400 ± 57.361 *
	5.loc	414.724 ± 9.434 **	554.594 ± 7.229 **	67.883 ± 0.323 *	716.575 ± 7.902 **	15,643.733 ± 64.542 *
B	Control	2.170 ± 0.055 **	2.697 ± 0.088 **	9.511 ± 0.093 **	11.558 ± 0.113 **	9.828 ± 0.097 **
	1.loc	18.915 ± 0.191 **	22.073 ± 0.157 *	15.578 ± 0.159 **	17.470 ± 0.112 *	90.587 ± 0.818 *
	2.loc	16.394 ± 0.264 **	20.299 ± 0.211 **	15.582 ± 0.154 **	15.558 ± 0.110 *	77.898 ± 0.529 *
	3.loc	25.316 ± 0.146 *	31.865 ± 0.136 *	15.272 ± 0.145 **	13.939 ± 0.141 **	97.970 ± 0.672 *
	4.loc	17.348 ± 0.134 *	28.897 ± 0.243 *	14.623 ± 0.117 *	11.688 ± 0.139 **	82.971 ± 0.192 *
	5.loc	15.833 ± 0.132 **	19.874 ± 0.118 *	15.888 ± 0.114 *	19.565 ± 0.119 *	71.414 ± 0.286 *
Ca	Control	4092.066 ± 47.394 **	4666.259 ± 76.230 **	2051.104 ± 45.618 **	2146.976 ± 73.989 **	23,075.947 ± 827.825 **
	1.loc	6228.160 ± 78.312 **	8118.432 ± 91.295 **	2140.800 ± 42.872 **	6720.480 ± 83.771 **	35,182.027 ± 89.263 *
	2.loc	5983.392 ± 72.422 **	7938.560 ± 91.866 **	2636.736 ± 63.722 **	6230.592 ± 58.320 *	32,482.080 ± 670.145 **
	3.loc	5816.928 ± 69.620 **	6530.208 ± 94.333 **	2250.880 ± 68.807 **	6632.672 ± 95.672 **	29,675.733 ± 754.383 **
	4.loc	8877.280 ± 101.416 **	9979.584 ± 117.985 **	2380.448 ± 72.292 **	8607.200 ± 81.906 *	40,611.307 ± 484.719 **
	5.loc	7895.392 ± 74.387 *	8420.032 ± 76.022 *	2469.632 ± 37.798 **	7697.984 ± 84.248 **	38,124.267 ± 966.413 **
Cd	Control	0.448 ± 0.015 **	0.571 ± 0.030	0.429 ± 0.009 **	0.499 ± 0.023	0.532 ± 0.018 **
	1.loc	1.289 ± 0.024 **	3.110 ± 0.123 **	1.899 ± 0.080	3.032 ± 0.067 **	5.594 ± 0.132 **
	2.loc	0.810 ± 0.036 **	2.542 ± 0.082 **	1.337 ± 0.052	2.113 ± 0.053 **	3.408 ± 0.077 **
	3.loc	1.004 ± 0.049	2.286 ± 0.048 **	1.264 ± 0.071	1.896 ± 0.093	4.120 ± 0.136 **
	4.loc	1.190 ± 0.082	3.015 ± 0.083 **	1.505 ± 0.053	2.489 ± 0.095 **	4.657 ± 0.149 **
	5.loc	1.299 ± 0.038 **	3.609 ± 0.058 **	2.384 ± 0.060 **	2.696 ± 0.079 **	4.664 ± 0.058 **
Cr	Control	3.315 ± 0.097 **	4.942 ± 0.106 **	2.073 ± 0.081 **	4.084 ± 0.125 **	10.964 ± 0.087
	1.loc	15.393 ± 0.127 **	23.041 ± 0.120 *	2.190 ± 0.080 **	3.006 ± 0.115	64.457 ± 0.239 *
	2.loc	13.268 ± 0.142 **	17.636 ± 0.114 **	1.711 ± 0.086	6.049 ± 0.157 **	52.018 ± 0.543 **
	3.loc	6.427 ± 0.110 **	12.138 ± 0.083 *	2.189 ± 0.078 **	3.031 ± 0.087 **	40.980 ± 1.114 **
	4.loc	7.554 ± 0.134 **	14.542 ± 0.086 *	4.891 ± 0.133 **	5.402 ± 0.151 **	34.567 ± 0.385 **
	5.loc	2.354 ± 0.074 **	3.434 ± 0.091 **	3.438 ± 0.139 **	6.668 ± 0.084 **	26.690 ± 0.238 *
Cu	Control	3.444 ± 0.111 **	4.011 ± 0.079 **	2.050 ± 0.082 **	5.366 ± 0.117 **	11.553 ± 0.088
	1.loc	17.217 ± 0.169 *	24.255 ± 0.190 *	18.634 ± 0.103 *	20.202 ± 0.180 *	65.801 ± 0.092 *
	2.loc	15.244 ± 0.111 *	19.667 ± 0.321 **	16.723 ± 0.139 *	25.894 ± 0.254 **	55.303 ± 0.142 *
	3.loc	20.365 ± 0.224 **	24.887 ± 0.231 **	18.379 ± 0.153 *	25.046 ± 0.173 *	76.221 ± 0.344 *
	4.loc	18.241 ± 0.160 **	28.013 ± 0.137 *	20.640 ± 0.202 **	24.976 ± 0.116 *	52.278 ± 0.145 *
	5.loc	21.893 ± 0.109 *	29.242 ± 0.165 *	18.336 ± 0.070 *	18.773 ± 0.126 *	62.200 ± 0.975 **
Fe	Control	144.775 ± 1.178 **	215.987 ± 4.067 **	65.981 ± 4.069	90.346 ± 1.233 **	5383.413 ± 28.453 *
	1.loc	1009.249 ± 7.515 *	1292.450 ± 5.638 *	226.782 ± 5.638 **	292.414 ± 5.763 **	7611.733 ± 46.187 *
	2.loc	795.834 ± 8.046 **	1276.728 ± 8.682 *	203.825 ± 8.682 **	353.107 ± 9.377 **	6766.080 ± 53.806 *
	3.loc	975.455 ± 9.169 **	1221.311 ± 17.208 **	251.115 ± 17.208 *	411.475 ± 8.997 **	7212.480 ± 62.371 *
	4.loc	1063.679 ± 17.107 **	1139.595 ± 7.658 *	233.864 ± 7.658 **	408.835 ± 10.245 **	8087.627 ± 41.110 *
	5.loc	957.339 ± 8.739 *	1085.740 ± 8.206 *	273.897 ± 8.206 **	381.409 ± 5.303 **	7355.947 ± 20.667 *
K	Control	6062.752 ± 68.309 **	6780.128 ± 87.108 **	4714.816 ± 72.447 **	5902.688 ± 111.560 **	25,884.107 ± 452.582 **
	1.loc	4958.533 ± 90.357 **	6188.640 ± 74.525 **	1674.083 ± 39.989 **	3372.814 ± 88.565 **	18,616.853 ± 173.420 *
	2.loc	4279.242 ± 89.226 **	5177.098 ± 62.050 **	2065.471 ± 59.532 **	3374.260 ± 74.245 **	15,920.053 ± 32.570 *
	3.loc	4103.054 ± 120.023 **	5628.185 ± 86.518 **	1853.015 ± 51.099 **	4079.966 ± 115.704 **	14,566.987 ± 55.416 *
	4.loc	5034.572 ± 112.770 **	5384.120 ± 118.745 **	1834.350 ± 55.609 **	3674.378 ± 76.546 **	21,982.453 ± 291.887 **
	5.loc	5009.806 ± 143.166 **	5538.193 ± 106.233 **	1715.235 ± 49.574 **	2605.313 ± 54.719 **	26,475.787 ± 533.518 **
Mn	Control	8.790 ± 0.231 **	11.011 ± 0.241 **	5.850 ± 0.171 **	28.874 ± 0.812 **	73.880 ± 0.264 *
	1.loc	72.841 ± 0.169 *	111.122 ± 0.394 *	9.106 ± 0.085 **	34.285 ± 0.116 *	226.752 ± 1.982 *
	2.loc	58.528 ± 0.233 *	90.555 ± 0.229 *	5.307 ± 0.116 **	58.258 ± 0.442 *	180.773 ± 7.094
	3.loc	81.866 ± 0.075 *	138.261 ± 0.149 **	10.452 ± 0.113 **	45.235 ± 0.346 *	197.640 ± 4.691 **
	4.loc	103.127 ± 0.235 **	171.730 ± 4.567 **	7.063 ± 0.137 **	39.063 ± 0.147 *	268.144 ± 2.632 **
	5.loc	86.134 ± 0.118 *	141.210 ± 0.123 **	7.330 ± 0.092 **	35.715 ± 0.114 *	219.625 ± 2.246 **
Mg	Control	895.264 ± 8.754 **	980.736 ± 12.003 **	314.304 ± 8.547 **	586.688 ± 13.950 **	2746.880 ± 18.938 *
	1.loc	1348.269 ± 9.894 *	1703.684 ± 36.420 **	330.944 ± 7.502 **	1598.240 ± 22.807 **	4227.253 ± 75.626 **
	2.loc	2081.376 ± 89.050 **	2582.272 ± 63.872 **	585.056 ± 3.958 *	1911.136 ± 84.761 **	3807.733 ± 35.542 *
	3.loc	708.160 ± 7.211 **	1051.424 ± 8.225 *	365.920 ± 7.333 **	1484.420 ± 9.548 *	2505.120 ± 69.097 **
	4.loc	1316.096 ± 6.551 *	1869.248 ± 53.782 **	267.552 ± 6.862 **	1880.384 ± 70.802 **	4135.733 ± 82.677 **
	5.loc	763.680 ± 8.292 **	983.424 ± 9.889 **	363.424 ± 5.119 **	1810.592 ± 52.500 **	2803.787 ± 67.939 **
Na	Control	149.782 ± 0.107 **	176.384 ± 0.930 *	93.427 ± 0.893 **	146.464 ± 1.028 *	1500.133 ± 2.750 *
	1.loc	424.457 ± 4.718 **	653.427 ± 5.244 *	169.066 ± 4.815 **	157.495 ± 0.669 *	2641.621 ± 25.832 *
	2.loc	311.096 ± 3.886 **	587.514 ± 4.288 **	217.400 ± 2.873 **	202.223 ± 5.565 **	2187.893 ± 36.462 **
	3.loc	719.830 ± 7.680 **	966.745 ± 4.360 *	247.384 ± 6.854 **	278.409 ± 6.265 **	3634.720 ± 15.954 *
	4.loc	680.365 ± 5.343 *	874.122 ± 6.223 *	264.201 ± 3.893 **	167.937 ± 5.054	3294.453 ± 9.398 *
	5.loc	521.448 ± 10.646 **	808.146 ± 4.977 *	215.326 ± 5.534 **	204.323 ± 7.998 **	3056.427 ± 55.255 **
Ni	Control	5.128 ± 0.116 **	6.758 ± 0.121 **	3.695 ± 0.117 **	5.735 ± 0.150 **	3.549 ± 0.028 *
	1.loc	17.845 ± 0.141 *	24.377 ± 0.116 *	11.919 ± 0.094 *	7.984 ± 0.082 **	48.088 ± 0.122 *
	2.loc	14.964 ± 0.093 *	21.381 ± 0.081 *	10.447 ± 0.116 **	6.204 ± 0.087 **	24.457 ± 0.354 **
	3.loc	17.345 ± 0.109 *	22.087 ± 0.093 *	11.652 ± 0.153 **	7.420 ± 0.114 **	30.071 ± 0.226 *
	4.loc	20.086 ± 0.144 *	23.410 ± 0.118 *	13.358 ± 0.118 *	9.913 ± 0.141 **	37.874 ± 0.296 *
	5.loc	21.385 ± 0.078 *	24.583 ± 0.201 *	15.097 ± 0.115 *	11.929 ± 0.143 **	42.254 ± 0.733 **
Pb	Control	2.375 ± 0.076 **	3.890 ± 0.112 **	3.709 ± 0.111 **	6.985 ± 0.141 **	26.142 ± 0.564 **
	1.loc	23.651 ± 0.203 *	30.155 ± 0.120 *	6.932 ± 0.117 **	14.638 ± 0.108 *	184.374 ± 8.229
	2.loc	24.288 ± 0.173 *	27.445 ± 0.128 *	5.864 ± 0.081 **	14.538 ± 0.075 *	190.234 ± 7.110
	3.loc	22.831 ± 0.164 *	36.911 ± 0.101 *	8.387 ± 0.140 **	16.560 ± 0.133 *	164.506 ± 2.810 **
	4.loc	15.599 ± 0.124 *	19.739 ± 0.115 *	5.019 ± 0.107 **	13.795 ± 0.091 *	135.776 ± 1.478 **
	5.loc	21.956 ± 0.208 *	24.730 ± 0.086 *	7.117 ± 0.108 **	14.609 ± 0.046 *	157.233 ± 2.107 **
Zn	Control	15.380 ± 0.233 **	16.767 ± 0.067 *	14.006 ± 0.182 **	12.880 ± 0.182 **	24.372 ± 0.185 *
	1.loc	82.655 ± 0.675 *	123.828 ± 0.496 *	58.764 ± 0.385 *	45.206 ± 0.400 *	263.242 ± 2.917 **
	2.loc	72.140 ± 0.078 *	105.077 ± 0.177 **	60.246 ± 0.343 *	86.646 ± 0.248 *	232.816 ± 4.490 **
	3.loc	90.128 ± 1.682 **	117.320 ± 0.535 *	59.426 ± 0.404 *	39.872 ± 0.129 *	302.480 ± 4.120 **
	4.loc	103.171 ± 0.226 *	151.209 ± 0.787 *	79.087 ± 0.320 *	62.717 ± 0.165 *	347.551 ± 1.391 *
	5.loc	106.547 ± 0.803 *	137.384 ± 0.469 *	65.546 ± 0.463 *	57.396 ± 0.212 *	356.309 ± 4.039 **

* Variance analysis and Tukey test are indicated (*p* < 0.01, significant). ** Variance analysis and Tukey test are indicated (*p* < 0.05, significant).

**Table 4:** Reference levels for the toxic and normal amounts of heavy metals in plants (mg kg^−1^).

Element	Normal Concentrations	Toxic Concentrations	FAO/WHO *
Cr	0.006–18	>100	2.3
Cu	5–30	20–100	73.3
Fe	2–250	400–1000	425.6
Mn	30–300	300–500	500
Pb	0.1–10	30–300	0.3
Zn	25–150	100–400	99.4
Cd	0.2–0.8	5–30	0.2
Ni	0.1–5	30	67

* Permissible limits of heavy metals according to [71].

**Table 5:** Element contents in *Calamintha nepeta* subsp. *glandulosa* (mg kg^−1^ ± SD) samples: comparison with previous literature.

Elements	Present Study (Washed Leaves, from Location 1)	*Calamintha baetica* from Barbadalhos mine/Portugal [73]	*Calamintha grandiflora* from Velebit/Croatia [74]	*Calamintha nepeta* from Setúbal District/Portugal [29]	*Calamintha nepeta* from Ordu/Türkiye [72]
Al	461.4 ± 5.4		118 ± 0.1		
Ag *		0.3 ± 0.1			
B	18.9 ± 0.2		24.9 ± 0.1	18.7 ± 2.4	
Ba *			54.8 ± 0.1		
Ca	6228.2 ± 78.3		12.164 ± 0.1	7862 ± 1224	
Cd	1.3 ± 0.1		0.037 ± 0.1		
Co *		0.93 ± 0.1	0.044 ± 0.1		
Cr	15.4 ± 0.1	0.66 ± 0.1	0.368 ± 0.1		
Cu	24.3 ± 0.2	16 ± 0.1	10.6 ± 0.1	9.25 ± 3.96	2.9± 0.3
Fe	1009.2 ± 7.5	287 ± 0.1	103 ± 0.1	264 ± 98	221.0 ± 50.4
K	4958.5 ± 90.4		454 ± 0.1	12110 ± 2430	
Mg	1348.3 ± 9.9		2730 ± 0.1	1843 ± 314	
Mn	72.8 ± 0.2		105 ± 0.1	198 ± 77.8	
Na	424.5 ± 4.7		257 ± 0.1		
Ni	17.8 ± 0.1	2.3 ± 0.1	0.648 ± 0.1		
Pb	23.7 ± 0.2	52 ± 0.1	4.86 ± 0.1		
S *					1454.4 ± 209.8
Zn	82.7 ± 0.7	90 ± 0.1	49.1 ± 0.1	52.7 ± 18.9	106.8 ± 7.3

* Elemental data for Ag, Ba, Co, and S are reported from literature sources only, as these elements were not analyzed in this work.

**Table 6:** EDI (mg/kg/day), THQ of Cd, Cr, Ni, and Pb, and HI values for adults * associated with consumption of plant samples.

	Cd		Cr		Ni		Pb		
Localities	EDI	THQ	EDI	THQ	EDI	THQ	EDI	THQ	HI
Control	5.31 × 10^−4^	1.77 × 10^−3^	3.30 × 10^−2^	1.65 × 10^−2^	2.46 × 10^−2^	1.64 × 10^−2^	2.54 × 10^−2^	2.54 × 10^−3^	0.037
1. loc	3.09 × 10^−3^	1.03 × 10^−2^	1.36 × 10^−1^	6.78 × 10^−2^	1.02 × 10^−1^	6.83 × 10^−2^	1.50 × 10^−1^	1.50 × 10^−2^	0.161
2. loc	2.13 × 10^−3^	7.11 × 10^−3^	1.14 × 10^−1^	5.72 × 10^−2^	7.39 × 10^−2^	4.92 × 10^−2^	1.51 × 10^−1^	1.51 × 10^−2^	0.129
3. loc	2.18 × 10^−3^	7.27 × 10^−3^	8.06 × 10^−2^	4.03 × 10^−2^	8.40 × 10^−2^	5.60 × 10^−2^	1.45 × 10^−1^	1.45 × 10^−2^	0.118
4. loc	2.67 × 10^−3^	8.90 × 10^−3^	8.56 × 10^−2^	4.28 × 10^−2^	9.88 × 10^−2^	6.59 × 10^−2^	1.09 × 10^−1^	1.09 × 10^−2^	0.128
5. loc	3.07 × 10^−3^	1.02 × 10^−2^	5.31 × 10^−2^	2.65 × 10^−2^	1.09 × 10^−1^	7.25 × 10^−2^	1.30 × 10^−1^	1.30 × 10^−2^	0.122

EDI: estimated daily intake; THQ: target hazard quotient; HI: hazard index; * 70 kg adult person.

**Table 7:** RDA (%) values for nutrients in the leaves of *Calamintha nepeta* subsp. *glandulosa* (mg).

		Ca	Cu	Fe	K	Mg	Mn	Na	Zn
	RDA	1000 (mg)	0.9 (mg)	8 (mg)	4700 (mg)	420 (mg)	2.3 (mg)	1500 (mg)	11 (mg)
Washed Leaf	Control	40.92	38.26	180.97	12.90	21.32	38.22	1.00	13.98
	1. loc	62.28	191.30	1261.56	10.55	32.10	316.70	2.83	75.14
	2. loc	59.83	169.38	994.79	9.10	49.56	254.47	2.07	65.58
	3. loc	58.17	226.28	1219.32	8.73	16.86	355.94	4.80	81.93
	4. loc	88.77	202.67	1329.60	10.71	31.34	448.38	4.54	93.79
	5. loc	78.95	243.26	1196.67	10.66	18.18	374.50	3.48	96.86
Unwashed Leaf	Control	46.66	44.57	269.98	14.43	23.35	47.88	1.18	15.24
	1. loc	81.18	269.50	1615.56	13.17	40.56	483.14	4.36	112.57
	2. loc	79.39	218.52	1595.91	11.02	61.48	393.72	3.92	95.52
	3. loc	65.30	276.52	1526.64	11.97	25.03	601.14	6.44	106.65
	4. loc	99.80	311.25	1424.49	11.46	44.51	746.65	5.83	137.46
	5. loc	84.20	324.91	1357.18	11.78	23.41	613.96	5.39	124.89

70 kg adult—based on 100 g dw^−1^ consumption per day.

## Data Availability

The raw data supporting the conclusions of this article will be made available by the authors on request.
